# Increasing Access and Availability of Nutrient-Dense Foods at United States Marine Corps Food Venues Is Feasible and Profitable

**DOI:** 10.3390/nu17152556

**Published:** 2025-08-05

**Authors:** Katie M. Kirkpatrick, Zina N. Abourjeily, Melissa A. Rittenhouse, Maureen W. Purcell, Rory G. McCarthy, Jonathan M. Scott

**Affiliations:** 1 Consortium for Health and Military Performance, Department of Military and Emergency Medicine, F. Edward Hébert School of Medicine, Uniformed Services University, 4301 Jones Bridge Road, Bethesda, MD 20814, USA; 2 Henry M. Jackson Foundation for the Advancement of Military Medicine, Inc., 6720A Rockledge Drive, Bethesda, MD 20817, USA

**Keywords:** tactical, military, military nutrition, dietary intervention, food environment, behavioral design

## Abstract

**Background/Objectives**: Military Service Members (SMs) require optimal nutrition to support health, readiness, and job performance. However, they often fall short of meeting nutrition guidelines. This study aimed to determine the impact and feasibility of implementing the U.S. Marine Corps (USMC) “Fueled to Fight^®^” (F2F) nutrition program in non-appropriated fund (NAF) food venues. Objectives included evaluating changes in Military Nutrition Environment Assessment Tool (mNEAT) scores, feasibility of implementing and maintaining F2F strategies, and influence on customer purchasing patterns. **Methods**: Researchers conducted a pre-post interventional study from January to December 2024 at three NAF food venues across two USMC bases. F2F strategies, including identifying items using a stoplight color coding system (Green = healthy, Yellow = less healthy, Red = least healthy), menu revisions, food placement, promotion, and marketing, were implemented. Data included mNEAT assessments, sales reports, and stakeholder focus groups. Generalized Estimating Equations models were used to analyze sales data. **Results**: mNEAT scores increased across all venues post-intervention. Availability and sales of Green items increased, while sales of Red items decreased in some venues. Profit increased at all three food venues. Focus groups revealed feasibility and provided insights for future interventions. **Conclusions**: F2F interventions in NAF food venues are feasible and can positively impact the food environment and customer purchasing patterns without negatively affecting profit. This study highlights the importance of integrating nutrition programs into all military food venues, not just government-funded dining facilities, to support the nutritional fitness and readiness of SMs.

## 1. Introduction

Military Service Members (SMs), like other tactical populations, require a high level of fitness to meet job demands, participate in training exercises, manage physical and mental stress, and cope with environmental factors. Optimal nutrition—which requires the provision and consumption of food in appropriate quantities, quality, and proportions to sustain mission performance and protect against disease and injury—is critical to SMs’ general health and readiness to perform their military duties. A nutritionally adequate diet with an appropriate calorie level, along with behavioral strategies and physical activity, are well-documented strategies for weight management [1]. Given their dietary patterns, SMs often do not meet nutrition standards, achieve optimal body composition, or engage in healthy eating behaviors, impacting their ability to reach optimal nutrition [2,3,4,5,6,7]. In addition, obesity has a negative impact on military recruitment and retention, musculoskeletal conditions, and mental health [8]. Overweight and obesity, based on body mass index, are associated with higher diagnoses of a wide range of medical conditions, including endocrine, nutritional, and metabolic diseases [9] that could prevent optimal health and readiness and negatively impact job performance.

To support the nutritional needs of SMs, military bases offer different food operations with a variety of venue types, locations, and offerings. Together, these offerings constitute the military nutrition environment: all food, beverage, and dietary supplement options available on a military base. There is high interest in optimizing Department of Defense (DoD) food programs to provide high-quality and cost-effective food service to the military community as evidenced by recent U.S. Government Accountability Office reports [10,11]. Nutrition is a key topic in other military quality-of-life initiatives, including strategies to help active-duty SMs to combat food insecurity [10,11,12]. Increased healthy food availability has shown to increase the odds of healthy food selection across individuals with both higher and lower socioeconomic positions, making it a practical population-level strategy [13].

Nutrition-related efforts to improve health often target improving food–environment components—physical (availability, quality, and promotion), economic (cost), policy, and sociocultural (norms and beliefs)—given their impact on consumer food selection [14]. These types of interventions are likely to be effective (including cost-effective) in reducing obesity and non-communicable diseases [14], in addition to reaching a larger audience than individually targeted interventions. Different food–environment interventions—such as modifying food quality or quantity, menu, or price; targeting food choices at point of purchase through labels, information, or education; and less often, implementing policy, either alone or in combination—have been explored in a variety of settings [15], with positive results.

Previous research reported that food–environment interventions in workplace settings positively impacted outcomes, including overall dietary intake (specifically increased intake of fruits and vegetables), improved health outcomes (e.g., weight loss, cholesterol reduction), nutrition knowledge, and sales of healthy foods [15]. Nutrition labeling is a common intervention that can be implemented in numerous ways (e.g., nutrition information, symbols, “healthy” labels). A 2021 review of post-secondary food–environment interventions revealed that overall nutrition labeling increased the consumption of healthy foods [16]. In addition, information strategies (food labels, posters) across different settings resulted in either an increase in sales of targeted foods or positive changes in eating patterns in most cases [17]. Specifically, worksite and university venues (cafeteria, vending machines) were noted as having the most potential for success [17]. Also, combining choice architecture interventions with nutrition labels yielded positive results [16].

Another intervention strategy, menu modification including increasing more nutritious items, decreasing less nutritious items, or both), goes beyond information-focused interventions to increase the availability of nutritious options, therefore decreasing reliance on diners to translate information to behavior. Worksite cafeteria menu modifications of entrees to decrease calories, saturated fat, and sodium and increase unsaturated fat and fiber led to increased sales and revenue [18]. A six-site cafeteria study that targeted swapping out less nutritious cooked meals, sandwiches, snacks, and cold beverages for more nutritious versions reduced calorie consumption without a negative impact on revenue [19]. Other research has shown that financial incentives (e.g., price reduction) can increase the sales of nutritious foods [16].

Similar strategies are in place at U.S. military bases to support health and readiness by building a supportive food environment that encourages access, availability, and awareness of nutrient-rich options. A 2024 review and meta-analysis of military food–environment interventions found that stoplight color coding, choice architecture, and menu-modification strategies improved overall diet quality and proportion meeting nutrient needs, while maintaining high levels of diner satisfaction [20]. Nutrition and menu standards differ among the types of military food service operations, driven by policy, contracts, and priorities. In appropriated-fund (money allocated from Congress) food venues, color-coded nutrition labeling programs such as Go for Green^®^ (G4G; named Fueled to Fight^®^ (F2F) in USMC) are required [21,22]. Specifically, USMC policy outlines requirements for F2F program execution and promotion [23]. Non-appropriated fund (NAF; non-government funded, for-profit) food venues are not required to follow appropriated fund policies and instead may execute their own nutrition programs (e.g., BeFit [24] and Better 4U) in convenience stores, snack bars, micro marts, vending machines, and other venues. These venues must generate profit; thus, efforts to improve the food environment by increasing the availability and accessibility of healthier—sometimes more expensive and less desirable—options are often seen as conflicting with the need to maintain profit margins.

G4G and F2F consist of eight program requirements that address the following strategies: training, menu revisions (including stoplight coding), food placement, food promotion, and marketing/education [25,26,27,28]. Research with 100 active-duty Soldiers showed Healthy Eating Index (HEI) scores improved significantly after G4G program implementation [29]. Diners significantly increased their selection of whole grains, seafood, and plant proteins and ate fewer refined grains. More diners agreed that main dishes were nutritious and performance-based and reported using color-code labels to choose performance foods [29]. Other research showed the positive impact of the G4G and F2F program components on diner selection. Specifically, menu and choice architecture interventions led to a decreased intake of calories, total fats, saturated fat, sodium, and refined grains among diners while maintaining or improving SM satisfaction [30,31].

Although the implementation of the G4G and F2F program (referred to as F2F moving forward) in traditional dining facilities has shown positive impact on diner behaviors, there is limited research about nutrition programs in NAF venues. In particular, the impact of interventions on sales and profit has not yet been explored within NAF venues. Incorporating F2F in NAF food venues would provide a consistent way for SMs to identify nutritious foods across a variety of food venues. Therefore, the purpose of this study was threefold: first, to determine the impact of implementing F2F strategies in NAF venues on pre- and post-intervention Military Nutrition Environment Assessment Tool (mNEAT; see below) scores; second, to evaluate the feasibility of implementing F2F strategies in NAF venues; and finally, to examine how F2F strategies influence customer purchasing patterns in NAF venues, specifically their impact on item sales and profit.

## 2. Materials and Methods

### 2.1. Study Sample

Researchers conducted a study from January to December 2024 to assess the feasibility and impact of implementing F2F program components at two USMC bases. The Uniformed Services University of the Health Sciences led the research with the U.S. Army Research Institute of Environmental Medicine acting as consultants. The research team proposed the study to the U.S. Marine Corps Community Services (MCCS), who support Marines and their families through a variety of programs and services, including various food venues. The study and proposed interventions supported USMC’s quality-of-life initiatives [32]. The study was approved by the Uniformed Services University of the Health Sciences Institutional Review. After obtaining the required approvals, there was a formal inquiry to seek a USMC base interested in participating. Ultimately, MCCS selected two bases with geographic proximity. MCCS leadership signed a letter of support for base participation, which outlined the expectations of both research and MCCS teams, study timelines, and points of contact.

MCCS selected a total of three food venues across the two bases—a micro mart, a set of three vending machines, and a snack bar—based on the following criteria: high-traffic and high-volume location, active-duty Marines as the primary customers, proximity to barracks, supportive management and staff, and one venue that supports off-hours population (Table 1).

**Figure A1:**
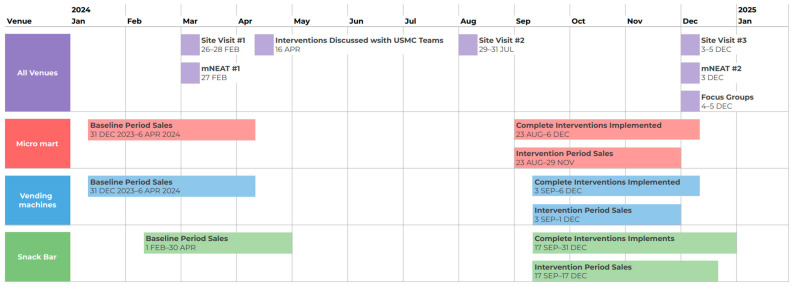
Timeline of study activities: site visits, mNEATs conducted, focus groups, and sales periods.

This study aimed to determine the impact of implementing F2F strategies in NAF venues on pre- and post-intervention Military Nutrition Environment Assessment Tool (mNEAT; see below) scores; to evaluate the feasibility of implementing F2F strategies in NAF venues; and to examine how F2F strategies influence customer purchasing patterns in NAF venues, specifically their impact on item sales and profit. Researchers assessed the impact and feasibility of F2F implementation at NAF venues using both quantitative and qualitative metrics. Data collection, observation of food venues and interventions, and engagement with stakeholders occurred at three site visits (February, July, and December 2024) throughout the study (see Appendix A, Figure A1 and Table A1). In addition, to collaborate efficiently and navigate challenges, researchers communicated weekly via email or video calls with MCCS and local teams.

### 2.2. Objective 1: Data Collection and Statistical Analysis

To address the first objective—determine the impact of implementing F2F strategies in NAF venues on pre- and post-intervention mNEAT scores—mNEAT was conducted at each of the three food venues at baseline and then 3 months later. mNEAT is a standardized, evidence-based DoD tool that assesses the quality of the food environment at different venue types across three key categories: food policy (policy, training), availability of healthy food (menu, food and beverage offerings), and behavioral design (food promotion, choice architecture, marketing/education). MCCS and researchers conducted the mNEAT during the first site visit to obtain a baseline score. Each venue was evaluated using the assessment appropriate to its venue type (see Appendix B.1, Appendix B.2 and Appendix B.3). Researchers, MCCS, and local food venue teams then collaborated to design interventions to improve the food–environment scores as measured by mNEAT, using F2F program components. The teams ensured that the interventions also aligned with food venue logistics, such as staffing, space, and resources. The research team provided intervention materials (i.e., posters, signage, window stickers, planograms) and F2F program resources (i.e., lists of pre-coded Green, Yellow, and Red items and standardized recipes) for the interventions. To determine the percentage of Green, Yellow, and Red, researchers assigned color codes to all food and beverages available at the three food venues using the F2F coding algorithm. F2F codes distinguish high-performance (Green), moderate-performance (Yellow), and low-performance (Red) foods and beverages according to evidence-based scoring components, the percentage of saturated fat, grams of fiber, grams of sugar, and degree of processing, and then comparing the total score to scoring ranges for each color code. The teams set a goal of increasing healthy (Green) items to 20%.

While the mNEAT assesses aspects of food availability, the cross-sectional nature of the tool does not always reflect the variation in availability throughout the day or week as is typical in food service. Thus, researchers used bi-weekly itemized sales reports from the micro mart and vending machines to determine typical availability of foods and drinks by Green, Yellow, and Red codes during baseline and intervention periods. Researchers assigned each item an F2F color code and an indication of where it was displayed (shelf, refrigerator, or freezer). When available, photos were used to determine whether items with zero sales across all seven bi-weekly periods were either unavailable (not visible in photos) or simply not purchased (visible in photos but not sold).

### 2.3. Objective 2: Data Collection and Statistical Analysis

To address the second objective—evaluate the feasibility of implementing F2F strategies in NAF venues—managers at all three venues completed bi-weekly check-in surveys during the intervention period to ensure interventions were executed and maintained. Researchers assessed the feasibility of the interventions by analyzing the ability of local food venue staff to implement training, menu, choice architecture, promotion, and marketing/education interventions. This included evaluating staff knowledge, inventory availability, adherence to planograms (correct placement of Green, Yellow, and Red items), empty display slots, and maintenance of promotional materials. Analysis of planogram adherence—the percentage of items correctly placed according to color code, excluding empty slots (overall and by color code, weighted by display size, excluding empty slots) and the percentage of empty slots—was conducted using photos from the beginning and end of the intervention. Researchers used data from 7 of 8 intervention displays (one freezer had incomplete data due to a mechanical outage) in the micro mart and vending machines for calculations. This was not done for the snack bar as most of the food was made-to-order, so planograms were not part of the intervention and therefore not calculated.

During the final site visit, the researchers also conducted three stakeholder focus groups (MCCS, and two food-venue teams—snack bar and vending/micro mart) to obtain food venue staff and leadership feedback. There were 2–9 participants per group for a total of 14 participants. To protect privacy, focus group participants were given a number, so their names, positions, and other personal identifiers were not recorded. Participants were informed they could decline answering any question or withdraw their participation at any time during the session. One researcher facilitated the session using the Institutional Review Board-approved set of questions, while the other researcher took notes. All sessions were audio-recorded. Researchers who were not involved present at the focus group sessions evaluated qualitative descriptions, an inductive approach to data analysis that identifies relevant themes as they emerge from the data, to review and assess information from the focus-group sessions [33] to further assess the feasibility of the interventions. Researchers used Rev.com [34], an artificial intelligence transcription service, to transcribe the audio files from the focus groups. Members of the research team then reviewed each transcript against audio recordings for accuracy. They identified and developed a codebook of key themes, which was then refined through an iterative process that involved testing codes against the data and developing agreed-upon code definitions. Once the codebook was finalized, all transcripts were co-coded using MAXQDA 2018 (VERBI Software. Consult. Sozialforschung GmbH, Berlin, Germany) qualitative analysis software and adjudicated for consensus.

### 2.4. Objective 3: Data Collection and Statistical Analysis

Lastly, to assess the third objective—examine how F2F strategies influence customer purchasing patterns in NAF venues (item sales and profit)—researchers collected and analyzed baseline and post-intervention sales data. Baseline was determined as the three-month period during which the first mNEATs were conducted, but before, researchers and stakeholders discussed potential interventions. Thus, for the micro mart and vending machines, the baseline was January–March 2024. The baseline for the snack bar was shifted one month (February–April 2024) because January 2024 sales reports were not available. The intervention period for all three venues was approximately September–November 2024—the first three months after all intervention strategies had been implemented. For both baseline and intervention periods, micro mart and vending machine managers provided seven bi-weekly itemized sales reports. The snack bar point-of-sales system could not provide itemized sales data; therefore, gross sales data in 13 weekly increments were used for both periods. For details on the frequency of metrics collected, see Appendix A, Table A1.

Using sales reports, researchers calculated the average bi-weekly availability and average bi-weekly profit of items by Green–Yellow–Red code during baseline and intervention periods. To better capture typical profit, they excluded one micro mart report (week of 7 October 2024) due to net negative sales and product loss from a freezer malfunction, and one vending machine report (week of 3 September 2024) because it covered only five days instead of the intended two weeks.

To investigate the impact of the interventions on item sales by color code in the micro mart and vending machines, researchers used a Generalized Estimating Equations (GEE) negative binomial with a log link model with SPSS Version 30 (IBM SPSS Statistics for Windows, IBM Corp., Armonk, NY, USA). This method is more robust for small sample sizes in a repeated measures analysis [35] than other methods, such as repeated measures ANOVA. Since the intervention occurred at the display level, the GEE treated the display as the unit of analysis, using an autoregressive covariance structure (AR-1) to account for dependencies over observation points. All displays (shelf, refrigerator, or freezer) with an intervention (5/7 at the micro mart; 3/3 at the vending machines) were included in the analysis, for a total of eight displays across the two venues. Three outcomes were modeled: the number of Green, Yellow, and Red items sold within the bi-weekly period while adjusting for the total number of items sold in the bi-weekly period. A dichotomous indicator was used for the intervention period (baseline period = 0; intervention period = 1) and the total number of items sold within the display for the bi-weekly period (to adjust for variance of sales across bi-weekly periods). The snack bar’s lack of itemized sales data did not allow for color-coded analysis. Instead, researchers calculated a weighted average by assigning weight to each week based on the number of days the snack bar was open to account for holiday and other closures. Weekly average weighted revenue and profit were calculated for the baseline and intervention periods. Statistical significance was set a priori at *p* < 0.05.

## 3. Results

### 3.1. Executed Food–Environment Interventions

Venues planned and executed interventions in all subcategories except policy (Table 2).

### 3.2. Impact of Food–Environment Interventions on mNEAT Scores

The food–environment scores of the three study venues were assessed at baseline and after a 3-month intervention using mNEAT. Post-intervention mNEAT scores increased across all three venues (Table 3). Behavioral design changes drove the largest total score increases (snack bar +8%; micro mart +10.8%; vending machines +100%), food availability contributed both increases and decreases (snack bar +8%; micro mart –1.4%; vending machines not applicable), and food policy made no contribution to changes in overall mNEAT scores as it was not attempted.

**Figure 1:**
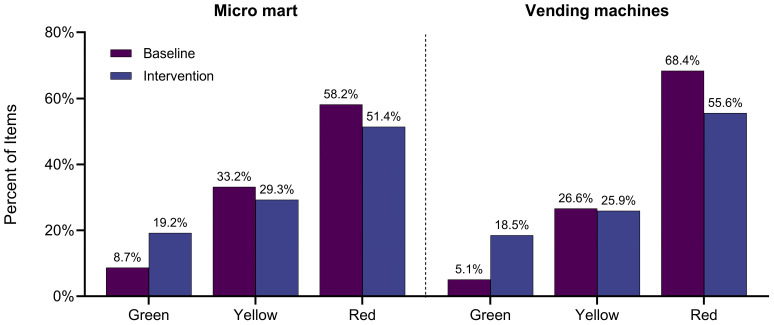
Average bi-weekly percentage of items available by F2F color code at the micro mart and vending machines: baseline vs. intervention.

A key component of food availability is the presence of healthy (Green) items for SM to choose from along with behavioral design elements to highlight them. Both the micro mart and vending machines had an increase in both the number and percentage of Green items offered, while the number and percentage of available Red items decreased (Figure 1). At the micro mart, the average bi-weekly percentage of unique Green items increased from 8.7% at baseline to 19.2% after intervention (from 17 to 40 items), while the number of Red items decreased from 58.2% to 51.4% (from 114 to 107 items). Similarly, at the vending machines, the average bi-weekly percentage of unique Green items increased from 5.1% to 18.5% (from 4 to 15 items), while the number of Red items decreased from 68.4% to 55.6% (from 54 to 45 items) (Figure 1). Following the intervention, the average bi-weekly availability of Green items approached the 20% target (micro mart = 19.2% and vending machines = 18.5%).

### 3.3. Feasibility of Food–Environment Interventions

One aspect of determining feasibility was adherence to choice architecture interventions, which compared recommended display setup versus executed display setup. At the start of the intervention, the overall average weighted planogram adherence was 78% but slightly decreased to 76% after three months. Adherence levels varied by item category: Green items showed the highest and most stable adherence (93–94%), followed by Red items (82–86%). Yellow items had the lowest adherence, remaining at 62–63% at both the beginning and end of the intervention period. The average percent of empty slots increased from 9% at the beginning to 15% at the end of the 3-month intervention period. Initially, the percentage of empty slots was similar across all color-coded food categories (8–9%). However, the average percentage of empty slots increased across all color codes after 3 months, with Green item slots having the highest percentage of empty slots (22%), followed by Yellow (18%), with Red slots having the lowest (11%).

Themes identified as a result of focus groups emerged around barriers and facilitators but focused on best practices and lessons learned. From a leadership viewpoint, MCCS felt communication was a barrier to greater success, and improvement could enhance future interventions. In addition, improved point-of-sales systems could more accurately analyze sales data. Local food venue teams described barriers to increasing food availability as difficulty identifying healthy (Green) items and properly labeling them. In addition, they noted challenges with obtaining a variety of healthy options from food vendors, with one participant stating “I think setup and execution were the easiest. I think the hardest part was … the variety and getting what we could use.” Local food venue teams described planograms as both a barrier (i.e., limiting flexibility of what was bought and where it was stocked) and a facilitator (i.e., quicker machine setup). They agreed that intervention success included buy-in from vendors and food venue managers. Local food venue teams felt interventions were feasible, and they offered various ideas for future intervention and expansion, including more diner education, the better identification of Green items, and the use of digital promotion (e.g., menu boards, television monitors, social media). Logistical suggestions included optimizing the timing of the project to earlier in the calendar year, selecting venues in higher-traffic areas, and improving sales report capabilities to optimize results and reach a broader audience.

### 3.4. Impact of Food–Environment Interventions on Purchasing Patterns

**Figure 2:**
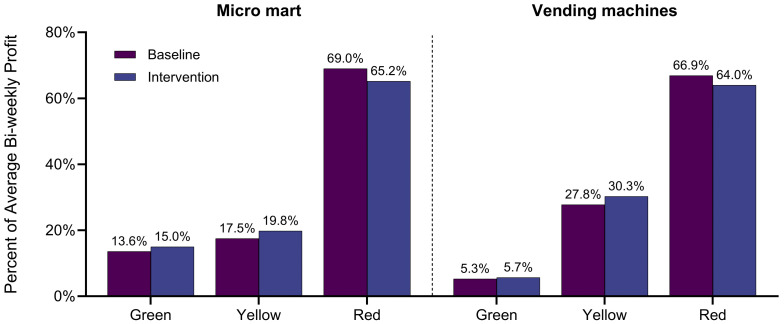
Percentage of average bi-weekly profit attributable to the sale items by F2F color: baseline vs. intervention.

**Figure A2:**
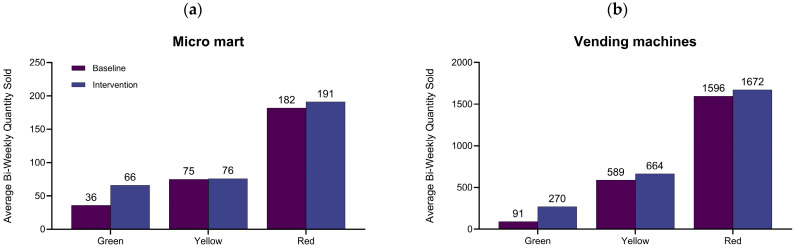
Average bi-weekly quantity of items sold by F2F color: baseline vs. intervention for (**a**) micro mart and (**b**) vending machines.

Average bi-weekly profit increased from baseline to intervention at both the micro mart (from USD 629.79 to USD 691.46) and vending machines (from USD 3238.96 to USD 3594.76), with profit increases observed across all color codes. The proportion of average bi-weekly profit attributable to Green, Yellow, and Red items shifted from baseline to intervention periods (Figure 2). At both venues, the percentage of profit from Green items slightly increased (from 14% to 15% at the micro mart; from 5% to 6% at the vending machines), while the percentage from Red items decreased (from 69% to 65% at the micro mart; from 67% to 64% at the vending machines). Profit from Yellow items also showed a slight increase at both venues. For information on the average bi-weekly quantity of items sold, see Appendix C, Figure A2.

Based on the GEE models, when all displays are included (n = 8), an average estimated 2.3 times more Green items were sold at each display during the intervention, accounting for temporal trends and items sold (Table 4; again, see also Appendix C, Figure A2).

At the snack bar, average weekly store revenue decreased by 8.05%, while overall profit increased by 4.15% during the intervention (Table 5).

## 4. Discussion

Researchers targeted three venues—a snack bar, micro mart, and set of three vending machines at two adjacent USMC bases—to improve the local food environment using F2F program components. Improvements indicated that the interventions positively impacted mNEAT scores, were feasible in the unique NAF settings that were targeted, and did not negatively impact profit. This study addresses a gap described in the 2024 Government Accountability Office report that despite representing a large part of the food landscape on military bases, NAF food venues have limited nutrition programs and initiatives [10,11]. In fact, recommendation 8 (of 16) from the report states, “The Secretary of Defense should ensure the Assistant Secretary of Defense for Health Affairs, in coordination with the forthcoming Defense Feeding and Nutrition Board, develops a strategy for increasing healthy menu options at non-appropriated fund food venues as part of its plan to increase access to healthy food on military bases” [11] (p. 50).

The impact of food–environment interventions can be a critical element to promote optimal nutrition and military performance. The Nutrition and Menu Standards for Human Performance Optimization regulation provides guidance on recommended nutrient intake tailored to the military population [21]. This policy is specific to appropriated-fund facilities, but NAF facilities can also support the nutritional needs of SMs by increasing the availability, accessibility, and awareness of nutritious food through standard evidence-based practices that are also customized to specific food venues, such as those executed in this study. A 2019 systemic review suggested public health policies and practices at multiple levels [36] to encourage the selection and consumption of nutritious foods, first to increase the proportion or number of healthy foods at the local level and also to mandate policy through nutritional criteria requirements to help ensure changes [36]. This is similar to executing the Nutrition and Menu Standards for Human Performance Optimization regulation (policy) and implementing the F2F program (local)—strategies that could be duplicated in NAF venues. Practices used in this study align with previous conclusions for effective interventions, including tailoring interventions to the population, management support and employee involvement, incorporating multiple components, and using a mixed-methods approach [37].

Food venues on military installations compete for the patronage of SMs and other customers, while SMs themselves must navigate competing demands and priorities. Previous military research exploring factors that impact SM food choices report the physical environment (such as availability and accessibility of foods), cost and convenience, time constraints, and proximity and density of fast-food outlets are barriers to nutritious eating [4,7]. One systematic review and meta-analysis of the food environment and obesity found that the proximity of fast-food outlets was associated with a higher risk of obesity. In contrast, the presence of supermarkets (with its range of healthier options) and fresh fruits and vegetables stores were associated with a lower risk of obesity [38]. This might be due, in part, to the availability of nutrient-dense options in these food venues compared to less nutritious options often found at fast-food restaurants.

The interventions executed during the USMC study led to increased availability of Green items at the micro mart and vending machines, with both venues offering 19% Green items, nearly reaching the 20% goal. This increase in Green items was critical because it paved the way for behavioral design interventions related to food promotion, choice architecture, and marketing/education. Previous research aligns with our findings, reporting increasing healthy item salience (the visibility and noticeability) alone and with educational information significantly increased fruit and vegetable intake and healthy food purchases [39]. In addition, the Food Service Guidelines for Federal Facilities outlines both food and nutrition standards, as well as behavioral design standards, many of which were used in this study [40], including several at the guidelines’ “innovative” (vs “standard”) implementation level. These guidelines highlight the feasibility of such efforts in various food venue settings and aligns with the U.S. Centers for Disease Control and Prevention Healthy Food Environments evidence-based strategies [41].

Given the variability in food venue operations, competing priorities, and available resources, offering and promoting more nutrient-dense options at a competitive price can be challenging. Overall, our qualitative and quantitative data demonstrated that the interventions executed during this study were feasible. Most of the local food venue teams and leadership involved had positive feelings about the progress made to improve the food environment, their involvement in the project, and implications for further impact and opportunities. This positive perception was corroborated by strong adherence to venue planograms and successful addition and promotion of Green items. Future efforts should ensure consistent availability of Green items throughout the day and across days, given the proportion of empty Green slots at the final site visit.

A common concern among food venue operators is the potential for healthy food–environment modifications to negatively impact sales. Sales data have been used as a metric in similar studies to assess the impact of interventions [16]. A previous study showed that 54% of studies reviewed reported significant increase in sales of healthy foods and drinks using stoplight labeling, healthy symbols, or price modification [15]. Specific to price modifications, a 2022 systemic review by Atanasova et al. included nine studies that reported price reductions positively impacted tracked and self-reported healthy item purchases [39]. For this USMC study, the interventions did not adversely affect profit at any of the food venues and, in fact, the percentage of profit from Green items slightly increased at the micro mart and the vending machines even with price reductions. Specifically, average bi-weekly profit at the micro mart and vending machines and average weighted weekly profit at the snack bar increased from baseline to intervention periods. Notably, the interventions had a statistically significant impact on the greater quantity of Green items sold at the micro mart and vending machines throughout the intervention period.

During focus groups’ sessions, local food venue teams felt that the more integrated these interventions become in the future, the larger the healthy food footprint becomes, and the greater the impact on the USMC community moving forward. Specifically, more venues participating in healthy food initiatives could lead to more efficient ways to procure and stock healthy items. Improving diet quality through increasing the availability and accessibility of nutritious foods has been identified as a goal for military nutrition research [42]. A food environment with a comprehensive and consistent approach to prioritizing health could greatly benefit Marines and similar populations in meeting nutrition standards, achieving optimal body composition, and encouraging healthy eating behaviors to support overall wellness and performance on the job and beyond.

### Limitations

This study had several limitations. First, researchers were unable to discern which intervention strategy (menu changes, choice architecture, price incentives, marketing) had the greatest impact because they were implemented concurrently. There were limitations in gathering metrics, including determining F2F color codes for all menu items (e.g., incomplete nutrition information, customization of certain made-to-order menu items) and mislabeling or misplacement of items by color code. For example, approximately 4–5 Yellow or Red items at the micro mart and vending machines were incorrectly labeled Green. Ideally, the baseline period to collect mNEAT scores and sales data could have been closer to the intervention period. Multiple factors were considered when initiating interventions based on food service operations (e.g., time required for collaborative efforts, procuring materials) and the local military population (e.g., annual relocation period for military members) and should continue to be considered in future research. In addition, there were limitations to tracking and analyzing sales data due to the point-of-sales system, fluctuations in customer traffic, and logistics impacting typical stocking and sales (e.g., holiday closures, seasonal fluctuations, freezers/refrigerators not working). The study design did not account for potential mediators and moderators, of which there were likely several. For example, moderators could have included fluctuations in base population, common in military settings, and personnel schedules (holidays/leave time, training, or pay) which could impact the numbers of customers and transactions. However, such variability is common in food service operations, and future research could explore these factors. Finally, the scope of the study did not include Marine and other customer feedback, which could have provided more information about the impact of interventions on purchasing patterns and nutritional knowledge.

## 5. Conclusions

Food–environment interventions have the potential to positively impact the eating behaviors of SMs who live, work, and train at military bases. This study has established a strong foundation to show the feasibility and impact of F2F interventions in NAF food environments, demonstrating the importance of involvement and buy-in from all levels. Strong leadership support, stakeholder engagement, and a shared goal of improving the food environment for Marines and other USMC community members were critical to the successful increase in and access to nutrient-dense food at three USMC food venues. Importantly, there were no adverse effects on profit across all three venues, suggesting that implementing food–environment interventions—including price decreases for Green items—can be financially sustainable and potentially beneficial for food service operations. Lessons learned and best practices can help inform future nutrition initiatives in USMC and other military branches and tactical populations to effectively enhance fueling for high-demand jobs.

## Figures and Tables

**Table 1:** Characteristics of selected food venues.

	Micro Mart	Vending Machines	Snack Bar
Description	Unattended market with drinks, snacks, and packaged frozen sandwiches and meals	One each for snacks, drinks, and cold items	Quick-service counter with made-to-order and prepared sandwiches and sides
Location	Office building also used for training classes	Barracks (Marine housing)	Co-located in high-volume Marine Mart (convenience store)
Population served	Employees and training class attendees (e.g., Marines, retirees)	Active-duty Marines	Active-duty Marines and other community members
Hours of operation	Monday–Friday 08:00–16:00	Monday–Sunday 00:00–24:00	Monday–Friday 06:00–16:00

**Table 2:** Executed interventions by mNEAT category and subcategory.

mNEAT Category	mNEAT Subcategory	Intervention Strategies	Micro Mart	Vending Machines	Snack Bar
Food policy	Policy	Not attempted			
	Training	Manager worked with staff to implement new interventions	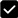	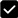	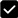
Food availability	Menu revisions	Increase healthy (Green) options	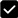	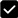	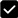
		Decrease unhealthy (Red) options	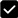	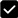	⬚
		Make healthy choice the default	⬚	⬚	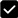
Behavioral design	Food promotion	Promote healthy items using print materials	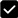	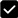	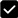
		Label healthy (Green) items with Green stickers at point of selection	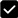	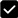	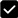
		Label section of healthy (Green) items with F2F window cling	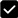	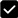	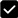
		Reduce price on most healthy (Green) items	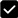	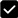	⬚
	Choice architecture	Move healthy (Green) items to eye level	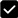	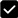	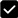
		Move unhealthy (Red) items to lowest display shelves	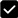	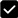	⬚
	Marketing/education	Promote venue using print materials outside of the venue	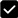	⬚	⬚

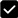
: An intervention was planned and executed; ⬚: No intervention was planned and executed.

**Table 3:** mNEAT scores at three venues pre- and post-intervention.

	Snack Bar		Micro Mart		Vending	
mNEAT Category	Pre	Post	Pre	Post	Pre	Post
Food Policy	0/2	0/2	1/4	1/4	n/a	n/a
Food Availability	7/15	9/15	6/26	5.5/26	n/a	n/a
Behavioral Design	1/8	3/8	0/7	4/7	0/3	4/4
Total	32%	48%	19%	28%	0%	100%

n/a = mNEAT assessment does not score food policy or food availability for vending.

**Table 4:** IRR and 95% CI of GEE models estimating the effect of intervention measures on the number of items sold by color in micro mart and vending (n = 8).

F2F Color Code	IRR (95% CI)	*p*-Value
Green	2.31 (1.36, 3.92)	0.002 *
Yellow	1.27 (0.97, 1.65)	0.08
Red	1.17 (0.93, 1.48)	0.172

* significant at *p*-value < 0.05; IRR: Incidence Rate Ratio; CI: Confidence Interval.

**Table 5:** Percent change in average (weighted) weekly snack bar revenue and profit: baseline vs. intervention.

	Baseline	Intervention	Percent Change
Average (weighted) store revenue	$23,814.85	$21,897.89	−8.05%
Average (weighted) profit	$10,682.38	$11,125.18	4.15%

## Data Availability

The data presented in this study are available on request from the corresponding author due to restrictions associated with data collection on military installations.

## Abbreviations

The following abbreviations are used in this manuscript:

SM	Service Members
G4G	Go for Green^®^
F2F	Fueled to Fight^®^
USMC	U.S. Marine Corps
NAF	Non-appropriated fund
mNEAT	Military Nutrition Environment Assessment Tool
MCCS	Marine Corps Community Services

## Appendix A

**Table A1:** Timing and frequency of metrics collected at baseline and intervention.

Venue	Period	3-Month, Bi-Weekly			Cross-Sectional	
		Itemized Sales Data	Gross Sales Data	Intervention Adherence Survey	mNEAT	Stakeholder Focus Groups
Micro mart	Baseline	☑	☑	⬚	☑	⬚
	Intervention	☑	☑	☑	☑	☑
Vending machines	Baseline	☑	☑	⬚	☑	⬚
	Intervention	☑	☑	☑	☑	☑
Snack bar	Baseline	⬚	☑	⬚	☑	⬚
	Intervention	⬚	☑	☑	☑	☑

☑: Data collected; ⬚: Data not collected.

## Appendix B

Three distinct mNEAT assessment types that matched the particular food venue were used at each food venue. The Express assessment form (B1) was used for the micro mart, the Vending Level 2 assessment form (B2) for the vending machines; and the Morale, Welfare, and Recreation assessment form (B3) for the snack bar.

### Appendix B.1

#### Express Assessment Form

FOOD POLICY
1. Is there a policy/guidance for inclusion of “healthy” foods and beverages in the store?
◯ Yes ◯ No
2. Is there a policy/guidance for placement and promotion of healthier items?
◯ Yes ◯ No
3. Is there a policy/guidance for use of a labeling identification program or procedure for labeling “healthier” items?
◯ Yes ◯ No
4. Is there a local practice to promote price adjustments and discounts for “healthier” foods and beverages?
◯ Yes ◯ No
FOOD AVAILABILITY
5. How many varieties of whole or pre-cut fresh fruit are available?
◯ 0 ◯ 1 –2 ◯ 3–4 ◯ ≥5
6. How many varieties of pre-cut, fresh vegetables are available?
◯ 0 ◯ 1–2 ◯ 3–4 ◯ ≥5
7. How many different options of low-fat yogurt with ≤30 g sugar/serving are available?
◯ 0 ◯ 1–2 ◯ 3–4 ◯ ≥5
8. How many options of dried fruit (no added sugar) and/or canned fruit (in its own juice or water) are available?
◯ 0 ◯ 1–2 ◯ 3–4 ◯ ≥5
9. How many additional healthier, grab-n-go protein snack options (tuna, chicken, hard boiled eggs, hummus, or peanut butter) are available?
◯ 0 ◯ 1 ◯ 2 ◯ ≥3
10. Are single serving/snack pack size (1–2 oz) available for varieties of chips, pretzels, etc.?
◯ Yes ◯ No
11. Are whole grain cereal options, ≤10 g sugar, and ≤230 mg sodium/serving available?
◯ Yes ◯ No
12. How many healthier frozen meal entrée options ≤500 cal and ≤600 mg of sodium are available?
◯ 0 ◯ 1–2 ◯ 3–4 ◯ ≥5 ◯ N/A
13. How many grab-n-go sandwich/wraps with a lean protein source/whole grain bread/wraps are available?
◯ 0 ◯ 1–2 ◯ 3–4 ◯ ≥5
14. How many grab-n-go salads, made with leafy greens and a lean protein, are available with no dressing added or dressing on the side?
◯ 0 ◯ 1–2 ◯ 3–4 ◯ ≥5
15. How many healthier hot food entrée or sandwich options (≤500 cal, ≤40% fat, 10% sat. fat), including breakfast sandwiches, are available?
◯ 0 ◯ 1–2 ◯ 3–4 ◯ ≥5
16. How many options of low fat (1%), nonfat milk, reduced fat choc. and other dairy alternative beverages are available?
◯ 0 ◯ 1 ◯ 2 ◯ ≥3
17. How many different options of 100% fruit and vegetable juice are available?
◯ 0 ◯ 1 ◯ 2 ◯ ≥3
18. How many healthy (≤25 cal/8 oz.) bottled beverage options are available?
*Do not include milk, juice, or diet soft drinks. Healthy beverages are those that have <25% of calories from (natural) sugar and has no added caloric sugar (sucrose, HFCS,* etc.*)*
◯ 0 ◯ 1–2 ◯ 3–4 ◯ ≥5 ◯ N/A
19. How many healthy (≤25 cal/8 oz.) fountain beverage options are available? This includes water, infused water, unsweetened ice tea etc.
*Do not include milk, juice, or diet soft drinks. Healthy beverages are those that have <25% of calories from (natural) sugar and has no added caloric sugar (sucrose, HFCS,* etc.*)*
◯ 0 ◯ 1–2 ◯ 3–4 ◯ ≥5 ◯ N/A
BEHAVIORAL DESIGN
20. Is there a grab-and-go area with healthy sandwich/wraps, salad, or snack options?
◯ Yes ◯ No ◯ N/A—no grab-n-go
21. Are there signs, banners, displays or posters encouraging healthy food, snacks, or drink choices?
◯ Yes ◯ No
22. At the checkout counter, are there healthier options (fresh fruit, baked chips, trail mix) available?
◯ Yes ◯ No
23. At the checkout counter, are healthier beverages available in the beverage cooler?
◯ Yes ◯ No ◯ N/A—no beverage cooler
24. Are healthier beverages positioned at eye-level in the cooler?
◯ Yes ◯ No ◯ N/A
25. Are low-fat dairy products placed at eye level in the cooler?
◯ Yes ◯ No ◯ N/A
26. Are endcap displays used for promotion of nutrition initiatives (Be-FIT, Better 4 U) and healthy eating ideas?
◯ Yes ◯ No
27. Are healthy food icons, characters/displays used throughout the venue to identify healthier food choices?
◯ Yes ◯ No ◯ N/A
28. Are there discounts, or specials promoting healthy food choices?
*For example: combo meals or coupons advertised at the pump*
◯ Yes ◯ No

### Appendix B.2

Vending Level 2 Assessment Form
1. Are “healthy” options labeled and identified as healthier selections?
*(Healthy is defined as a minimum standard of FitPick Workplace)*
◯ Yes ◯ No
2. Are the healthier options/FitPick items placed in the center locations (Center, eye level) in the machine?
◯ Yes ◯ No
3. Are the healthier options/FitPick items placed in the correct and properly labeled slots in the machine?
◯ Yes ◯ No ◯ N/A
4. Is there messaging/graphics on/near the vending machines to promote healthy eating/healthy lifestyles?
◯ Yes ◯ No

### Appendix B.3

#### Morale, Welfare, and Recreation Assessment Form

FOOD POLICY
1. Is there a headquarters or local policy to include healthy food and beverage options (as an example: at least 25% of the full menu, minus alcohol) at the venue? Ask the manager this question.
◯ Yes ◯ No
2. Is there a policy to identify healthier options on menu boards/menus? Ask the manager this question.
◯ Yes ◯ No
FOOD AVAILABILITY
3. How many healthier a la carte main meal options (meat or vegetarian), including salads, are available?
Count each option separately, except low fat deli meats counts as 1 option.
*Healthier main meal options include: skinless chicken/turkey breast, low-fat deli meat (chicken, turkey, ham), veggie/turkey burgers, fish/seafood, pork top loin, beef top round steak or roast, items that is not prepared by breading or frying, and is not covered in sauce or salad dressing*
*Healthier items are described as: baked, grilled, sautéed, stir fried, steamed*
◯ 0 ◯ 1–2 ◯ 3–4 ◯ ≥5
4. Are healthier options listed first for their category on the menu board or menu?
*Example: Healthier side items (such as fresh fruit) is listed first in the “side” section*
◯ Yes ◯ No
5. How many different healthier side options are available? Side is defined as a single serving of a food that may accompany a meal or entree or eaten on its own.
*Healthier side options include: prepared side salad (not from a salad bar), fruit without added sugar/syrup, baked/steamed vegetables, legume (beans) without added fat, brown/wild rice, quinoa, couscous, barley, and or whole grain pasta.*
*Healthier items are described as: baked, grilled, sautéed, stir fried, steamed*
◯ 0 ◯ 1–2 ◯ 3–4 ◯ ≥5
6. How many vegetable variety options are available for salad options?
*Can be fresh, chopped/diced vegetables or fruit, or canned beans*
◯ 1–4 ◯ 5–8 ◯ 9–12 ◯ ≥13 ◯N/A—no salads
7. How are high fat sauces, condiments, and/or salad dressing served?
*High fat sauces, condiments, salad dressing include cream-based, cheese-based, butter-based, or mayonnaise-based sauces.*
*Do not include ketchup, mustard, avocado*
◯ Placed automatically on the dish ◯ Served on the side automatically
◯ Served on the side by request ◯ N/A—No condiments available
8. How many whole grain bread/tortilla wrap options are available?
*Whole grain options include: wheat, oats, barley, rye*
◯ 0 ◯ 1 ◯ 2 ◯ ≥3
9. How many healthier grab-and-go snack options are available?
*Healthier Grab-n-Go option can include:*
*Granola/protein bars with low-sugar or no sugar added (<200 kcals)*
*Plain nuts/seeds and/or dried fruit/nut trail mixes without added sugar, candy, or chocolate (<200 kcals)*
*Dried fruit without added sugar (<200 kcals) and/or canned/packaged fruit in its own juice or water*
*Baked pre-packaged snacks (chips, pretzels) (<200 kcals)*
*Hummus, hard-boiled eggs, fresh veggies, whole fruit,* etc.
◯ 0 ◯ 1–3 ◯ 4–7 ◯ 7–10 ◯ ≥11 ◯ N/A—no grab & go available
10. How many different healthy beverage options are available?
*Healthy beverages are defined as:*
*Beverages with ≤25 calories per 8 oz service (exclude fruit juice and milk)*
*Water, including flavored, carbonated, and seltzer water*
*100% juice—fruit or vegetable juice with no added sugar*
*Fat free or 1% milk (both flavored or unflavored)*
*Fortified soy, almond, or rice milk with <12 g sugar per serving*
◯ 0 ◯ 1–5 ◯ 6–10 ◯ 11–15 ◯ ≥16
BEHAVIORAL DESIGN
11. Are healthier items promoted outside the entrance (on the door, windows, or freestanding signs) so that you see the health messaging before you walk into the establishment?
◯ Yes ◯ No
12. Inside the establishment, are there table tents, posters, or signs on the wall that promote healthier options?
◯ Yes ◯ No
13. Are there healthier options (fresh fruit, baked chips, packaged plain nuts or seeds, trail mix) available at the point of service?
◯ Yes ◯ No ◯ N/A—sit-down restaurant
14. Are healthier beverages positioned at eye-level in the beverage cooler?
◯ Yes ◯ No ◯ N/A—no beverage cooler
15. Are healthier menu options identified (as “healthier” or with a logo) on menu boards or menus?
◯ Yes ◯ No
16. With meal, can you substitute the less healthy default (e.g., fries or chips) for a healthier option (e.g., side salad or fruit) at no cost? NA = No combo meals
◯ Yes ◯ No ◯ N/A
17. Is health messaging/promotion of healthier substitutions emphasized and highly visible at the point of service? (e.g., highlighted on the menu, menu board, and/or sign by point of sale)
◯ Yes ◯ No
18. Is the calorie information clearly visible (next to menu items) to customers?
◯ Yes ◯ No
19. Is additional nutrition information (more than calories) available upon request by the customer?
*Either on the website, in a handout, or booklet/binder*
◯ Yes ◯ No
20. Are the “healthier” items advertised as specials of the day or “chef’s specials”?
◯ Yes ◯ No

## Appendix C

In order to calculate the average bi-weekly quantity of items sold by F2F color at baseline and intervention, only the eight displays used for the GEE model were used. In addition, to better capture typical quantity of items sold, researchers excluded one micro mart report (week of 7 October 2024) due to net negative sales from a freezer malfunction and product loss, and one vending machine report (week of 3 September 2024) because it covered only five days instead of the intended two weeks.
